# Expression Analysis of Long Non-Coding PCAT-1in Breast Cancer

**Published:** 2017-07-01

**Authors:** Shaghayegh Sarrafzadeh, Lobat Geranpayeh, Soudeh Ghafouri-Fard

**Affiliations:** 1 Department of Medical Genetics, Shahid Beheshti University of Medical Sciences, Tehran, Iran; 2 Department of Surgery, Sina Hospital, Tehran University of Medical Sciences, Tehran, Iran

**Keywords:** Breast cancer, lncRNA, PCAT1

## Abstract

**Background:** The prostate cancer-associated non-coding RNA transcript 1 (PCAT-1) is a newly identified long non- coding RNA whose participation in tumorigenesis of a variety of cancers has been observed. In the present study, we aimed at analysis of its expression in breast cancer patients.

**Materials and Methods:** The expression of PCAT-1 was assessed using real-time reverse transcription polymerase chain reaction in tumor samples obtained from 47newly diagnosed breast cancer patients as well as their corresponding adjacent non-cancerous tissues (ANCTs).

**Results:** We detected significant over-expression of PCAT-1 in 12/47 (25.5%) of tumoral tissues compared with their corresponding ANCTs. However, no significant association has been found between the levels of PCAT-1 transcripts and patients’ clinical data such as tumor size, stage, grade, estrogen and progesterone receptors or Her2/neu status.

**Conclusion: **PCAT-1 is possibly involved in the pathogenesis of fraction of breast cancers. Future studies are needed to evaluate its precise function in breast cancer.

## Introduction

 Breast cancer is the most common cancer among women^   1 ^. It has been regarded as a complex and heterogeneous disease distinguished by histological parameters as well as clinical markers such as hormone receptor status. Although this classification has been rather successful in determination of patient’s prognosis, a high level of disparity has been detected in patient’s response to treatment modalities. Consequently, gene expression profiling has been suggested as an efficient modality for classification of breast cancer subtypes, which can show the different cellular origins and degree of progression^        2 ^. Several previous studies have evaluated the expression of protein-coding genes in breast cancer tissues in an attempt to find novel cancer biomarkers or therapeutic targets 3^,^^4^ . However, recent studies have shown that the human genome comprises many thousands of long non-coding RNAs (lncRNAs), which are produced through pathways comparable with that of protein-coding genes with similar histone-modification profiles, splicing signals, and exon/intron lengths^   5 ^. This part of human transcriptome has gained attention of researchers in human cancer because lncRNAs have potential to be applied as cancer biomarkers as well as therapeutic targets ^6^^-^^9^ . Prostate cancer-associated IncRNA transcript 1 (PCAT-1) is among lncRNAs which has been found by high-throughput sequencing of poly A+ RNA (RNA-Seq) from a cohort of prostate tissues and cells lines^        10 ^. This lncRNA has been shown to have a prostate-specific expression profile with minimal expression detected by quantitative real-time PCR in breast or lung cancer cell lines^   11 ^* . *PCAT-1 is a transcriptional repressor that participates in the regulation of cell proliferation and serves as a target of the Polycomb Repressive Complex 2 (PRC2). Notably, the researchers have suggested a method for categorization of patients into molecular subtypes based on the patterns of PCAT-1 and PRC2 expression^        10 ^. This lncRNA has been found to regulate BRCA2 expression in the post-transcriptional level^        12 ^. Furthermore, a prostate cancer risk-associated variant at rs7463708 has been shown to increase binding of a recently identified androgen receptor (AR)-interacting transcription factor, at a distal enhancer that loops to the PCAT-1 promoter, which leads to over-expression of PCAT-1following continued androgen treatment. Besides, PCAT1 cooperates with AR and LSD1 and is necessary for their gathering at the enhancers of two androgen late-response genes involved in prostate cancer tumorigenesis^10^. On the other hand, the AR has been demonstrated to be expressed in 3 main breast cancer subtypes. AR has a significant interaction with estrogen receptor (ER),so its participation in different signaling pathways is probably different among breast cancer subtypes^   13 ^. So, PCAT-1 may contribute in the pathogenesis of breast cancer as well. Furthermore, elevated expression of PCAT-1 has been shown to be associated with decreased BRCA2 expression in prostate cancer cells^        12 ^. Considering the role of BRCA2 in the repair of double strand breaks (DSB) and the contribution of its mutations in the pathogenesis of a fraction of breast cancers, PCAT-1 expression analysis would pave the way for identification of a mechanism for deficiencies in DSB repair in patients lacking BRCA mutations. In addition, PCAT-1–mediated proliferation in prostate cancer cells has been shown to be exerted through cMyc protein stabilization. In other words, PCAT-1 has a protective effect on cMyc by disruption of MYC regulation by miR-34a^        14 ^. Consequently, in the present study, we evaluated the expression of PCAT-1 and its relation with MYC transcript levels in a population of Iranian breast cancer patients.

## MATERIALS AND METHODS

**Patients' samples**

Forty-seven newly diagnosed female breast cancer patients referred to Sina and Farmanieh Hospitals in 2015 were included in the present study. All patients were diagnosed with invasive ductal carcinoma of breast. Patients with history of cancer in other organs or history of radio or chemotherapy were excluded from the study. Tumoral tissues in addition to their ANCTs were obtained from all patients during surgery under the conventions of the Ethics Committee. ANCT was defined as the normal breast tissue diagnosed by the pathologists through H.E. staining. Informed consent was obtained from all study participants. Clinical and pathological information of patients were collected through questionnaires and assessment of medical records. The samples were immediately snap-frozen in liquid nitrogen and then stored at -70⁰C.

**RNA extraction and quantitative real-time reverse transcription polymerase chain reaction (RT-PCR)**

Total RNA was extracted from tissue samples by the means of the AccuZol™ total RNA extraction solution (Bioneer, Korea) based on the manufacturer’s protocol, except for an extended 1- hour treatment with DNase. Thermo Scientific NanoDrop™ 1000 Spectrophotometer was used for the assessment of RNA purity and concentration. Then, PrimeScript RT reagent kit (Takara Bio, Ohtsu, Japan) was applied for cDNA synthesis using one µg of RNA. Quantitative real-time reverse transcription polymerase chain reaction (RT-PCR) was implemented on a Rotor-Gene 6000 from *Corbett* detection system using SYBR Premix Ex Taq (Takara Bio, Ohtsu, Japan). PCR protocol consisted of an initial activation step for 10 minutes at 95º C, followed by 40 cycles at 95 ºC for 15 seconds, specific annealing temperature for 15 seconds and 72 ºC for 20 seconds. The specific annealing temperatures were 62 ºC for PCAT-1 and beta 2 microglobulin (B2M) and 56.5 º C for MYC. In addition, each run contained no template control (NTC) consisting of H_2_O. B2M gene was used asnormalizer. Specific primers were designed using Primer-BLAST online tool^   15 ^. Forward and reverse primers sequences are as follows, respectively: PCAT-1: 5’- GAGAAGAGAAATCTATTGGAACC-3’ and 5’-GGTTTGTCTCCGCTGCTTTA-3’; B2M: 5’-AGATGAGTATGCCTGCCGTG-3’ and 5’-GCGGCATCTTCAAACCTCCA-3’; MYC: 5’-CACATCAGCACAACTACG-3’ and 5’-GTTCGCCTCTTGACATTC-3’. Melting curve analysis was performed to verify the specificity of the PCR products. Moreover, PCR products were electrophoresed on 2% agarose gel to verify product size and specificity.

**Estrogen receptor (ER), progesterone receptor (PR), Her2/neu and Ki-67 status**

The results of immunoreactivity for these markers were obtained from patients’ medical records. All of these markers were evaluated by immunohistochemical (IHC) staining. For ER and PR, staining of >20% of tumor cell nuclei was regarded as positive. For Her2/neu, a test result of 0 to 2+ was considered as negative and a result of 3+ as positive. Ki-67 values were stated as both the proportion of positively stained tumor cells amongst the whole number of tumor cells and as positive vs. negative. 

**Statistical analysis**

LinRegPCR(2) and Relative Expression Software Tool-RG©-version 3 (QIAGEN, Korea) were applied for calculation of fold changes in gene expression. The amplification efficiencies and cycle thresholds were included in the analyses. The quantities of mRNAs in the tissues were normalized to the B2M mRNA and compared between tumor and non-cancerous tissues. The pairwise fixed reallocation randomization test with 2000 iterations in the REST 2009 software was applied for the assessment of significance, which was set at the level of P<0.05. Besides, the correlation between PCAT-1 and MYC expression levels in a certain type of samples (tumor or ANCTs) was evaluated by the ratio of target genes over B2M expression (relative expression, REx). Data were analyzed using Pearson correlation statistical analysis. PCR assays were performed in duplicate for each sample, and the results were averaged. 

Statistical analyses of demographic and clinical data were performed using SPSSv.21 (SPSS Inc., Chicago, IL). The McNemar test was used to compare paired tumor and ANCTs. Chi-square and independent t tests were used to evaluate the association between categorical variables. The level of statistical significance for the p value was set at 0.05.

## Results

**General statistical information**

Patients’ information was obtained from questionnaires, interviews and evaluation of clinical and laboratory tests. Table 1 summarizes the demographic and clinical data of patients. 

**Table 1:** Demographic and clinical data of patients

**Age (mean±SD)**	**51.35 ± 14.721 (23 – 84)**
Menarche age (mean±SD)	13.41 ± 1.117 (12-16)
Menopausal status	
Premenopausal	56%
Postmenopausal	44%
Menopause age (mean±SD)	49.84 ± 4.413 (38 – 59)
First pregnancy age (mean±SD)	21.41 ± 5.056 (14-34)
Breast feeding duration (months) (mean±SD)	50.20 ± 52.206 (0-240)
Positive family history for cancer (%)	25.6
Body mass index (%)	
<18.5	7.1
18.5-24.9	57.1
>25	35.7
History of oral contraceptive use (%)	
Yes	67.4
No	32.6
History of hormone replacement therapy after menopause (%)	
Yes	18.6
No	81.4
Cancer stage (%)	
I	7.0
II	58.1
III	30.2
IV	4.7
Grade (%)	
I	11.9
II	54.8
III	33.3
Tumor size (%)	
<2 cm	16.3
≥2 cm, <5 cm	76.7
≥5 cm	7.0
Estrogen receptor (%)	
Positive	70.7
Negative	29.3
Progesterone receptor (%)	
Positive	63.4
Negative	36.6
Her2/neu expression (%)	
0	26.8
1	22.0
2	26.8
3	24.4
Ki67 expression (%)	
Positive	94.7
Negative	5.3

**Expression of PCAT1 in patients’ samples**

**Figure 1:**
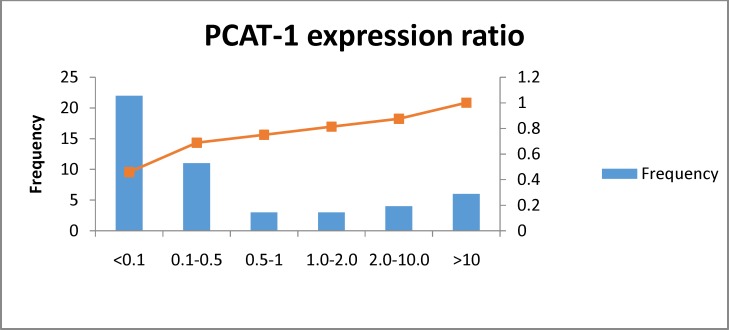
Frequency and cumulative percentage of samples in each subgroup based on relative expression of PCAT-1 in tumoral tissues compared with their corresponding adjacent non-cancerous tissue

**Figure 2:**
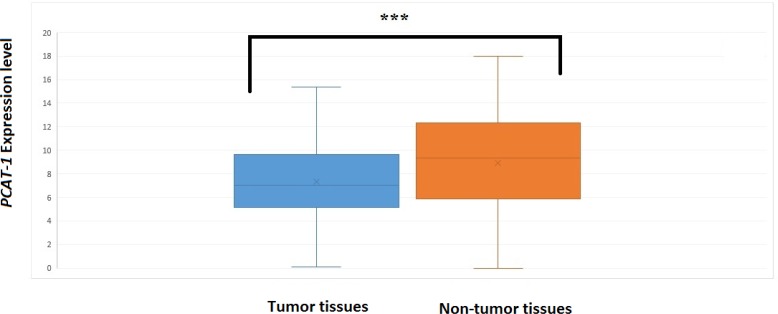
Relative expression of PCAT-1 in tumor tissues and adjacent non-cancerous tissues

We detected significant over-expression of PCAT-1 in 12/47 (25.5%) of tumoral tissues compared with their corresponding ANCTs. The relative expression values of PCAT-1 in these patients have been presented in Figure 1(the last three columns indicate the REx values more than 1). However, the level of PCAT-1 transcript was significantly lower in tumoral tissues compared with total tumor and ANCT tissues (P=0.02) (Figure 2). The frequency and percentage of patients' samples in certain subgroups based on relative expression of PCAT-1 in tumor tissues compared with ANCTs are demonstrated in Figure 1. MYC was shown to be over-expressed in all samples compared with their corresponding ANCTs (P<0.001). The comparison of the level of PCAT-1 transcripts with those of MYC showed no significant association (P=0.461).

The values under each column show the relative expression (REx) of PCAT1 in tumoral tissues compared with their corresponding adjacent non-canceroustissues.The X-axis shows the number of samples in each group based on REX values (left panel) and cumulative percentages (right panel).

**Correlations between genes expressions and clinical characteristics**

To define the role of PCAT-1 in breast cancer pathogenesis, we then evaluated the association between its transcript levels and several clinicopathological parameters. However, no significant associations were detected betweenPCAT-1 expression and these characteristics (Table 2). Among all patients, 2were negative for ER, PR and HER2/neu expression (triple negative). In the tumoral tissues of both patients, PCAT-1 was significantly down-regulated compared with their ANCT counterparts.

**Table 2:** PCAT-1 expression and its associations with clinical and demographic data

**Characteristics**	**Down regulation (relative expression<1)**	**Up regulation** **(relative expression>=1)**	**N**	**P**
Age				0.361
<50	18	7	25	
>=50	17	4	21	
Stage				0.897
0	1	0	1	
I	3	1	4	
II	20	6	26	
III	10	3	13	
IV	1	1	2	
Histological Grade				0.792
I	4	2	6	
II	19	5	24	
III	11	4	15	
Family History				0.330
No	24	9	33	
Yes	11	2	13	
Tumor Size				0.663
<2	7	1	8	
≥2 cm, <5 cm	25	9	34	
≥5 cm	2	1	3	
Node Status				0.250
Negative	22	5	27	
Positive	13	6	19	
ER Status				0.641
Negative	9	3	12	
Positive	24	8	32	
PR Status				0.435
Negative	12	3	15	
Positive	21	8	29	
Her2/neu Status				0.237
Negative	27	7	34	
Positive	6	4	10	
Ki67 Status				0.535
Negative	2	1	3	
Positive	30	8	38	
Hormone Replacement Therapy				0.635
Negative	28	9	37	
Positive	7	2	9	
Body Mass Index				0.130
18.5-24.9	1	2	3	
25-29.9	21	4	25	
>30	12	5	17	
Smoking History				0.569
Negative	33	10	43	
Positive	2	1	3	
Menopausal status				0.316
Premenopausal	18	8	26	
Postmenopausal	14	4	18	

## Discussion

 In the present study, we demonstrated significant up-regulation of PCAT-1 in a proportion of breast cancer samples compared with their paired ANCTs. To the best of our knowledge, the present study is the first report on expression analysis of this lncRNA in samples from breast cancer patients. A previous study has shown its minimal expression in breast cancer cell lines^   11 ^. Previously, it has been demonstrated that PCAT-1 expression in prostate cancer cells leads to a functional insufficiency in homologous recombination through its inhibitory effect on the BRCA2 tumor suppressor. The mentioned experiment has provided an explanation for the identification of impaired DSB repair mechanisms in cancers lacking BRCA1/BRCA2 mutations^        12 ^. Similar in vitro studies in breast cancer cell lines are needed to identify PCAT-1 role in DSB repair in breast cancer. In addition, PCAT-1 has been shown to be significantly over-expressed in about two-thirds of colorectal cancer tissues compared with the matched normal tissues. A significant association has been found between its expression and distant metastasis as well as poor overall survival^        16 ^. A more recent study demonstrated its up-regulation in hepatocellular carcinoma tissues compared with adjacent non-tumor tissues with a notable association between its relative transcript levels and TNM stage as well as metastasis^   17 ^. Another study has provided evidence for its contribution in lung cancer pathogenesis by showing that PCAT-1 suppression in these cells inhibits cell proliferation, migration and invasion, whereas its over-expression has the opposite effect^   18 ^. However, in the present study, we detected PCAT-1 over-expression in about only one-quarter of breast cancer tissues compared with their paired ANCTs, which implies that it only participates in the pathogenesis of this fraction of breast tumors. Furthermore, analysis of several clinicopathological data failed to show any association between levels of PCAT-1 transcripts and tumor characteristics, which can be at least partly explained by relatively small sample size of the study. On the other hand, we could not detect any correlation between the levels of MYC and PCAT-1 transcripts in the breast cancer tissues examined. Although PCAT-1 has been shown to stabilize cMyc level in prostate cancer cells, it has been shown to exert such effect post-transcriptionally by abolishing the down-regulation of cMyc by miR-34a^        14 ^. So, in vitro functional studies are needed to explore the role of PCAT-1 in regulation of MYC expression in breast cancer tissues. 

The contribution of rs7463708 as a prostate cancer risk-associated polymorphism in enhancing prostate transformation has been shown to be mediated through PCAT-1 up-regulation^        10 ^. On the other hand, the role of this variant in breast cancer risk has not been assessed yet. Future studies can focus on evaluation of any association between PCAT-1 transcript levels, the certain variant of this polymorphism and breast cancer risk. 

## CONCLUSION

 In brief, although lncRNAPCAT-1 has been shown to participate in many aspects of tumor progression, such as cell proliferation, migration and invasion in certain types of cancer^   18 ^, its up-regulation has only been detected in a fraction of breast cancers. So for its application as a therapeutic target in breast cancer, it is necessary to assess its expression in individual samples to predict its contribution in the tumorigenesis and to select the best treatment modality as stated in personalized cancer medicine.
